# The Rasch Analysis of Rosenberg Self-Esteem Scale in Individuals With Intellectual Disabilities

**DOI:** 10.3389/fpsyg.2019.01992

**Published:** 2019-09-06

**Authors:** Ju-Young Park, Eun-Young Park

**Affiliations:** ^1^Department of Rehabilitation, College of Medical Sciences, Jeonju University, Jeonju, South Korea; ^2^Department of Secondary Special Education, College of Education, Jeonju University, Jeonju, South Korea

**Keywords:** Rosenberg Self-Esteem Scale, Rasch, intellectual disabilities, psychometric properties, validity

## Abstract

**Background:**

The purpose of this study was to examine the psychometric properties of the Rosenberg Self-Esteem Scale for individuals with intellectual disabilities (ID) using the Rasch model and to determine whether the scale is valid and reliable for use with this population.

**Methods:**

Rasch analysis was carried out on data from 223 respondents to the 8th Panel Survey on Employment for the Disabled conducted by the Korea Employment Agency for the Disabled. The validity and reliability of scale items were verified through analyses of item fit, item difficulties, the rating scale, and separation indices.

**Results:**

Item infit mean square values were found to range between 0.71 and 1.25, and item outfit mean square values between 0.71 and 1.26. Additionally, item difficulties were appropriate; Item 4 was the most difficult item, while Item 10 was the easiest item. The 4-point rating scale was appropriate, and the separation indices were at an acceptable level.

**Conclusion:**

Based on these results, the validity and reliability of the Rosenberg Self-Esteem Scale for use with individuals with ID were verified. Thus, this scale can be regarded as a useful tool for evaluating the level of self-esteem of individuals with ID.

## Introduction

According to Rosenberg (1965a), self-esteem is one’s positive or negative attitude toward oneself and one’s evaluation of one’s own thoughts and feelings overall in relation to oneself. Self-esteem is regarded as a personal psychological characteristic relating to self-judgment based on one’s values about humans (Alesi et al., 2012). Self-esteem implies an awareness of one’s value system and one’s emotional evaluation of one’s self-worth (Schunk, 1985). High self-esteem indicates a high level of social adjustment (Martin et al., 2014). An individual with high self-esteem and an individual with low self-esteem may respond similarly to positive input, but they could exhibit different responses to negative input. Specifically, people with low self-esteem tend to exhibit negative responses, while those with high self-esteem tend to be less affected, as they are inclined to reject or restrict the scope of negative feedback (Brown and Mankowski, 1993). Because self-esteem affects an individual’s success in social adaptation, reduced self-esteem can eventually lead to social maladjustment. The relationship between low self-esteem and social adjustment could create a vicious cycle that negatively affects the overall quality of life (Konrad et al., 2012). In addition, self-esteem is a critical factor in personal well-being because an individual’s self-esteem has a positive relationship with their psychological health, social adjustment, and quality of life (Boyd et al., 2014). Self-esteem has also been reported to have a significant association with body image and has been used as a criterion to confirm the validity of other measures (Alesi and Pepi, 2016). As such, self-esteem is important on both personal and social axes in a social environment in which people live alongside others.

The Rosenberg Self-Esteem Scale (Rosenberg, 1965b) is the instrument most commonly used to measure self-esteem and has been used in a number of fields and has demonstrated comparable stability in many cultures. Schmitt and Allik (2005) studied the use of the Rosenberg Self-Esteem Scale, which was translated into 28 languages, in 53 countries and confirmed that it could be universally used in multiple cultures despite differences in some cultural characteristics. However, the unidimensional structure of the Rosenberg Self-Esteem Scale has been identified by some researchers as a problem with the measure (Martín-Albo et al., 2007). Some studies support a single dimensionality (Rosenberg, 1965b; Crandall, 1973; McCarthy and Hoge, 1982), while other findings have suggested a two-dimensional structure (Shahani et al., 1990; Hagborg, 1993). Furthermore, although the unidimensionality of the Korean version of the scale has been reported in the general population (Lee et al., 2009), its structure has not yet been reported for people with intellectual disabilities (ID). Although the Rosenberg Self-Esteem Scale is well referenced and researched in the literature and has generally demonstrated sound psychometric properties (Fleming and Courtney, 1984; Curbow and Somerfield, 1991) based on classical test theory, we were interested in determining how the item-level psychometrics provide information on the self-esteem of individuals with ID. As the total score of the Rosenberg Self-Esteem Scale was used to examine the participants’ level of self-esteem, unidimensionality was a prerequisite to use the total score as the measurement results.

### The Rosenberg Self-Esteem Scale and Intellectual Disability

According to American Association on Intellectual and Developmental Disabilities (AAIDD), intellectual disability is characterized by significant limitations in both intellectual functioning and adaptive behavior, which covers many everyday social and practical skills, and appears before the age of 18. Limitations in intellectual functioning often include difficulties with memory recall, task and skill generalization, and individuals with ID may demonstrate a tendency toward low motivation and learned helplessness. Issues in adaptive behavior may involve problems with abilities that affect day-to-day functioning in three areas, conceptual skills, social skills, and practical skills. Further, individuals with ID also often exhibit deficits in self-determination skills, including skill areas such as decision-making, problem-solving, and goal setting (Schalock et al., 2012). South Korea has adopted a classification system for individuals with ID based on IQ and adaptive behaviors. Grade 1 is defined as those with the most severe disability, indicating a person with an IQ of less than 35 who has significantly more difficulty adapting to everyday life and social life and needs protection for a lifetime. Grade 2 refers to a person with an IQ of 35–49 who can be trained to master simple behaviors in daily life. Grade 3 represents the least severe disability; it refers to a person with an IQ of 50 to 70 who does not require by some degree of supervision and help, who can hold a job that does not require special skills, and who is capable of social and occupational rehabilitation through education.

Research on self-esteem in individuals with ID has been conducted with individuals across the lifespan, including children, adolescents, adults, and the elderly. Research areas have included measurement and comparison of self-esteem levels, the effect of intervention programs on self-esteem, and social factors affecting self-esteem (Stanković and Milačić-Vidojević, 2014; Bana et al., 2017; Verberg et al., 2019). Kim et al. (2016) reported that self-esteem is correlated with leisure satisfaction and that those with high self-esteem report higher life satisfaction. Bana et al. (2017) reported that self-esteem significantly increased in a cognitive-behavioral play therapy group. Paterson et al. (2012) investigated the relationships between stigma, social comparison, and self-esteem in 43 adults with ID, and observed that perceived stigma is significantly associated with low self-esteem. Thus, existing studies have illustrated that the Rosenberg Self-Esteem Scale is a commonly used scale for the measurement of self-esteem in individuals with ID.

Previous studies of self-esteem in individuals with ID as measured using the Rosenberg Self-Esteem Scale have explored several research questions. This scale was used by Kim et al. (2016) in the exploration of leisure satisfaction, self-esteem, family satisfaction, and life satisfaction described above and by Paterson et al. (2012) in the study of stigma, social comparison, and self-esteem in adults described above. Additionally, it has been used in a study of the relationships between self-concept, self-esteem, and psychiatric symptoms in adults with ID by Garaigordobil and Pérez (2007), and by Dagnan and Sandhu (1999) in a study of social comparison, self-esteem, and depression in adults with ID.

### Assessments of the Psychometric Validity of the Rosenberg Self-Esteem Scale

Although the Rosenberg Self-Esteem Scale is one of the most popular measurement tools, its validity even in the general population has been debated. The unidimensionality of the Rosenberg Self-Esteem Scale has been found to be dependent on the characteristics of the population sampled, such as gender and grade level, and two distinct underlying factors have been identified in some research (Hagborg, 1993). Hagborg (1993) found a gender difference, with women reporting significantly lower RSE scores and a modest correlation between the total score and grade level. Another study that investigated the Rosenberg Self-Esteem Scale in several diverse samples reported that the scale mapped onto a one-dimensional construct and that this one-dimensionality is modulated by effects associated with the inclusion of negatively worded items (Corwyn, 2000). Analyses using item response theory (IRT) have also reported that the scale provides a highly reliable and internally consistent measure of global self-esteem (Gray-Little et al., 1997). Further, the underlying structure of the Rosenberg Self-Esteem Scale has been supported for use with participants in the US (Sinclair et al., 2010). However, questions remain as to whether this measure is structurally and psychometrically equivalent across cultures, languages, and groups (Schmitt and Allik, 2005).

Some psychometric variation across populations has been identified in existing research. Research aiming to verify the psychometric properties of the Rosenberg Self-Esteem Scale has been carried out across diverse groups, such as individuals with specific diseases or disabilities (Davis et al., 2009), people in different countries (Schmitt and Allik, 2005; Martín-Albo et al., 2007), those of both genders (Sinclair et al., 2010), and people of different ages (Hagborg, 1993). Schmitt and Allik (2005) reported a mean internal consistency of 0.81 with the lowest internal consistency in the Congo (0.45) and the highest internal consistency in Israel (0.90) and England (0.90). Sinclair et al. (2010) evaluated psychometric properties for the Rosenberg Self-Esteem Scale using a sample of US adults (*n* = 503), both overall and across demographic subgroups and reported that overall Rosenberg Self-Esteem Scale scores varied significantly across age, racial and ethnic, education, employment status, income, and marital status groups. Hagborg (1993) reported an internal consistency of 0.89 in 150 adolescents randomly selected from grades 8 to 12. Martín-Albo et al. (2007) reported that the internal consistency of the translated scale was assessed twice, and the Cronbach’s alpha was 0.85 and 0.88 at the two assessments, respectively. Further, these results showed that the value of the test-retest correlation was 0.84 and identified a one-factor structure in a sample of 420 university students.

These questions related to the scale’s dimensionality and reliability also apply to its use in populations of individuals with ID. Davis et al. (2009) reported on the psychometric properties of the Rosenberg Self-Esteem Scale, including a two-factor structure and moderate internal reliability, in a sample of 219 participants with ID. Moreover, the two-factor structure identified through factor analysis may be unsupported by IRT analysis. In a German general population sample, Roth et al. (2008) reported that confirmatory factor analysis showed the two-factor structure, while IRT analyses support a one-dimensional structure of the Rosenberg Self-Esteem Scale. However, there have been no studies that examined the psychometric properties of the Rosenberg Self-Esteem Scale in individuals with ID based on IRT as existing research on its psychometric properties in samples of individuals with ID has only utilized factor analysis (Davis et al., 2009).

One of the differences between Classical Test Theory and the IRT is item property invariance. The item constancy invariance refers to the item difficulty and item discrimination, which are the characteristics of the item, that are not changed by the characteristics of the subject group. It is possible to evaluate this with IRT, as it is analyzed using an item characteristic curve with unique characteristics of each item. The most frequently used method of IRT is the Rasch model, which evaluates the appropriateness of the item’s suitability and item difficulty (Rasch, 1960). Rasch analysis can analyze the difficulty and discrimination of each item, as well as estimate the real ability of the subject based on the analysis result. Further, Rasch analysis is advantageous in that the item characteristic estimation is not influenced by the characteristics of the target group (Cho, 1996; Kim et al., 1998). It is logical to analyze each item when verifying the appropriateness of the tool since the item is the most basic unit of the scale. Thus, IRT results systematically and logically evaluate item relevance, as it assesses the completeness of the test and the need to remove or modify items more stringently than classical test theory. Since the advent of IRT, studies that verify the psychometric properties of instruments have utilized IRT. Moreover, scales that have been previously standardized using Classical Test Theory have been revalidated using IRT (Kim and Park, 2011). One representative example is the Beck Depression Inventory. Although numerous validity studies have been conducted on the Beck Depression Inventory (Beck et al., 1979; Beck and Steer, 1984), the results of IRT showed that the validity of the confirmatory factor analysis alone did not adequately verify the structure of the dimensions and the difficulty of each item (Beck et al., 1988). To date, the one example of item-level psychometric evaluation of the Rosenberg Self-Esteem Scale was its verification in an elderly sample (Classen et al., 2007).

A strength of Rasch analysis, which is based on IRT, is that it can be used to estimate the real ability of a participant based on the results of the analysis (Chae et al., 2018). The present study aimed to identify the psychometric properties of the Rosenberg Self-Esteem Scale in individuals with ID using Rasch analysis. Using this method, the construct validity, reliability, item difficulty, and rating scale appropriateness could be verified in individuals with ID.

## Materials and Methods

### Sample

In order to test the psychometric properties of the Rosenberg Self-Esteem Scale in individuals with ID, data were taken from the 8th PSED (Panel Survey on Employment for the Disabled), provided by the Korea Employment Agency for the Disabled. The purpose of this panel survey is to provide basic data useful for the establishment and evaluation of policies on employment of people with disabilities by identifying relevant personal and environmental factors and producing basic statistics on overall economic activity over time. In the present study, we used data from the second wave of the survey completed in 2016. The criteria for inclusion in the survey were that participants were individuals with disabilities who were registered as such under the Welfare Act for Disabled Persons in Korea and who were between the ages of 15 to 64 years. South Korea had adopted a disability registration system. For registration, the diagnosis of the disability by a doctor is needed. Therefore, we limited participation in this study to those who were diagnosed with an ID by a doctor. We used the criteria defined by the AAIDD for ID which is characterized by significant limitations in both intellectual functioning and adaptive behavior, covering multiple everyday social and practical skills, with an onset before the age of 18. In South Korea, only registered individuals with disabilities could have eligibility for welfare service. After registration, services to support daily living, employment, welfare, pension payment, and tax reductions were provided by the government to individuals with disabilities.

This study is a secondary analysis of previously collected and publicly available data, and therefore, ethics approval was not required as per applicable institutional and national guidelines and regulations. The dataset could be found in the Korea Employment Agency for the Disabled web site^1^. The survey was carried out in accordance with Korean laws regarding the conduct of surveys. Participants provided informed consent to participate in the panel survey before data collection was conducted. If participants did not agree to complete the survey, the interview was not conducted. At the time of the initial investigation, the investigator provided each participant with a copy of the Confidentiality Document and Privacy Statement according to Statistical law.

For the sample design, the population list of persons registered with the Ministry of Health and Welfare was set as the population. The two-phase sampling method was adopted in which the number of extracted regions was adjusted, and an appropriate number of samples for each type of disability, disability grade, and age were extracted. A sample of the first phase disability was extracted through one step colony extraction method to extract regions, and stratified by the stratification based on the type of disability, disability grade, and age, and the stratification was extracted at a level satisfying the target error. The total number of panel participants was 4,577, of whom 398 were individuals with ID. Among panel participants with ID, data from 223 respondents were included in the present analysis; the rest were excluded because the data had been obtained from family members (166 participants) or there were missing data (9 participants). The general characteristics of the participants are shown in Table 1.

**TABLE 1:** Demographic characteristics of participants (*N* = 223).

**Category**	**Number**	**%**
**Gender**		
Male	139	62.3
Female	84	37.7
**Age**		
15–29	101	45.3
30–39	62	27.8
40–49	29	13.0
≥50	31	13.9
**Degree of disability**		
Grade 1	40	17.9
Grade 2	75	33.6
Grade 3	108	48.5
**Education level**		
Below elementary school	46	20.6
Middle school graduate	48	21.5
High school graduate	117	52.5
Above college	12	5.4
**Marital status**		
Married	30	13.5
Unmarried	193	86.5
**Basic living security recipient**		
Yes	95	42.8
No	127	57.2
**Employment**		
Yes	64	28.7
No	159	71.3
**Cognitive ability**		
Cognizant of time, place, and person	159	71.3
Partial cognizance of time, place, and person	58	26.0
No recognition of any of time, place, and person	6	2.7

*Grade 1, individuals with ID were those who were assessed to have an IQ less than 35 and a markedly difficult adaptation to everyday and social life, and requiring lifetime supervision. Grade 2, individuals with ID who had an IQ 35–49 and could be trained for tasks that required constant supervision or special skills to perform. Grade 3, individuals with ID were those who were assessed to have an IQ of 50 to 70 and were capable of social and occupational rehabilitation through education.*

The sample included 139 men (62.3%) and 84 women (37.7%). The most frequent age range was the 15–29 age group (*n* = 101, 45.3%) and the least frequent age range was the 40–49 age group (*n* = 29, 13.0%). The sample’s mean age was 33.22 (*SD* = 12.17) with a median age of 32. Regarding the degree of disability, Grade 3 individuals with ID were those who were assessed to have an IQ of 50 to 70 and were capable of social and occupational rehabilitation through education was the most common (48.4%), followed by Grade 2 individuals (33.6%) who had an IQ 35–49 and could be trained for tasks that required constant supervision or special skills to perform, and the fewest participants were rated as Grade 1 (17.9%), which indicated an IQ less than 35 and a markedly difficult adaptation to everyday and social life, and requiring lifetime supervision. High school graduates were the most common educational level (*n* = 117, 52.5%) followed by junior high school completion (*n* = 48, 21.5%). Most participants with ID were unmarried (*n* = 193) compared to 30 married participants. In terms of the receipt of benefits, most were non-recipients. More participants were unemployed compared to employed. Finally, the majority of participants (*n* = 159) were cognizant of time, place, and person.

### Measures

The PSED consisted of an eight-part questionnaire that included information about the panel, the status of participant’s economic activity, job ability, attitude of employment/environments, daily life/satisfaction of life, and general characteristics of the household that included the participant. There was no time limit to respond, and most interviews took about 1 h.

The Rosenberg Self-Esteem Scale (Rosenberg, 1965b) was administered as daily life/satisfaction of life part of the PSED. When the Rosenberg Self-Esteem Scale was translated in Korean, researchers made judgments about the content validity of each item in the original English version for its relevance to self-esteem, and there were no major changes in the original content or the translated version (Jeon, 1974). The Rosenberg Self-Esteem Scale consisted of 10 items, of which six were positive (Items 1, 2, 4, 6, 7, and 8), and four were negative (Items 3, 5, 9, and 10). The example of positive item was “I feel that I’m a person of worth, at least on an equal plane with others.” The example of negative item was “All in all, I am inclined to feel that I am a failure.” All items are shown in Table 2. Negative items were reverse-scored prior to analysis. The Rosenberg Self-Esteem Scale uses a 4-point response scale (1 = *strongly disagree*; 2 = *disagree*; 3 = *agree*; 4 = *strongly agree*). Cronbach’s α was 0.709 in this study.

**TABLE 2:** Item fit characteristics (*N* = 223).

**No.**	**Item content**	**Measure**	**SE**	**Infit**		**Outfit**	
					
				**MNSQ**	***Z*-value**	**MNSQ**	***Z*-value**
1	I feel that I’m a person of worth, at least on an equal plane with others.	52.37	1.08	0.71	–3.60	0.71	–3.50
2	I feel that I have a number of good qualities.	45.97	1.12	0.83	–1.90	0.78	–2.40
3	All in all, I am inclined to feel that I am a failure.	47.22	1.11	1.17	1.80	1.16	1.60
4	I am able to do things as well as most other people.	58.31	1.06	1.15	1.70	1.17	1.90
5	I feel I do not have much to be proud of.	54.56	1.07	1.12	1.30	1.09	1.00
6	I take a positive attitude toward myself.	52.49	1.08	0.81	–2.20	0.80	–2.30
7	On the whole, I am satisfied with myself.	56.05	1.07	0.72	–3.50	0.71	–3.60
8	I wish I could have more respect for myself.	47.22	1.11	1.18	1.80	1.13	1.30
9	I certainly feel useless at times.	45.47	1.13	1.07	0.80	1.06	0.60
10	At times I think I am no good at all.	40.36	1.16	1.25	2.40	1.26	2.40

*Measure, item difficulty, which refers to the difficulty of endorsing an item and is expressed on a logit scale; MNSQ, mean square; SE, standard error; the possible range of MNSQ was from 0 to infinity; the expected MNSQ values for all items are 1.0.*

Demographic information that was collected included gender (male, female), age (aged 15–29 years, aged 30–39 years, aged 40–49 years, aged above 50 years), educational level (below elementary school education, middle-school graduate, high-school graduate, graduated college or higher), disability level (categories), degree of disability (Grade 1, Grade 2, Grade 3), marital status (living with a spouse, others), receiving basic living security benefits (yes, no), employment status (employment, unemployment), and cognitive ability. Cognitive ability comprised three categories related to a sense of time, place, and person, and being cognizant of all three was coded as “1,” partial cognizance of all three was coded as “2,” and no recognition of the three was coded as “3.”

### Statistical Analysis

Rasch analysis was performed using WINSTEPS, version 3.61.2 (Linacre, 2006b), to calculate the psychometric properties of the Rosenberg Self-Esteem Scale, including item fit, item difficulties, rating scale, and separation index. First, the rating scale model in Rasch was selected for calculating the parameters (Andrich, 1978):

$$\mathrm{Pr}\left\{X_{n\,i}=x\right\}=\frac{\exp\,\sum_{k=0}^{x}\left(\beta_{n}-\left(\delta_{i}+\tau_{k}\right)\right)}{\sum_{j=0}^{m}\exp\,\sum_{k=1}^{j}\left(\beta_{n}-\left(\delta_{i}+\tau_{k}\right)\right)}$$

Where δ_*i*_ is the difficulty of item *i* and τ_*k*_ is the *k*th threshold location of rating scale, which is in common to all the items, *m* is the maximum score and is identical for all the items, β_*n*_ is the location of person *n*, and τ_*o*_ is chosen for computational convenience. The variable *X*_*ni*_ is a random variable that can take on integer values between 0 and a maximum. *Pr*⁡{*X*_*ni*_ = *x*} means that the probability of the outcome *X*_*ni*_ = *xis*. Estimates of item difficulty (δ_*i*_) and person’s self-esteem status (β_*n*_) were expressed on a logit scale.

#### Item Fit

Internal scale validity, which is also referred to as item fitness, indicates how closely the actual responses to items match the expected responses based on the Rasch model. The judgment is based on the infit mean-square (MnSq) and outfit MnSq values. Infit MnSq values represent anomalous responses to items at the competency level of the participant, and outfit MnSq values represent anomalous responses to items outside the competency level of the participant. When the infit or outfit MnSq is below 0.5 or above 1.7, the item fit is considered unacceptable (Bond and Fox, 2013). The closer the item fitness score to 1, the more fully the item reflects the construct being measured (Waugh and Chapman, 2005). In the case of an MnSq value greater than 1.7, the item is regarded as a misfit indicating that it does not reflect the construct. When the MnSq value is less than 0.5, this represents an unacceptable overfit (Bond and Fox, 2013) and suggests that there is a high likelihood that the item is a duplicate of other items. Misfit or outfit items should be reviewed and corrected or removed from the scale.

The formula for calculating infit MnSq was:

$$\mathrm{infit}\,\mathrm{MnSq}=\sum_{n}^{N}W_{n\,i\,Z^{2}\,n\,i}/\sum_{n}^{N}W_{n\,i}$$

Where *W*_*ni*_ is variance of *x*_*ni*_, and $Z_{n\,i}^{2}$ is standardized residual squared. Infit MnSq weights the observations by variance. Infit is less influenced by outliers and more sensitive to patterns of inlying observations.

The formula for calculating the outfit MnSq was:

$$\mathrm{outfit}\,\mathrm{MnSq}=\sum_{n=1}^{n}Z_{n\,i}^{2}/N$$

Where $Z_{n\,i}^{2}$ is standardized residual squared (Wright and Masters, 1982). Outfit MnSq is based on the sum of squared standardized residuals. Outfit is more sensitive to unexpected observations by person who are scoring the items.

#### Item Difficulty

Individual participant attributes and item difficulty were compared using the distributions of item scores and the participants’ scale scores, which were graphed together according to each respective attribute score in order to enable direct comparison. As individual attribute scores and item difficulties were each converted to a logit scale, it was possible to make a direct comparison. When the ranges of the two different distributions are consistent (i.e., when the distribution ranges of the item difficulties are similar enough that the item difficulties reflect all ranges of the individual attribute scores and difficulties), the distribution is considered adequate (Chae et al., 2018).

#### Rating Scale

The rating scale function represents the ability of the participant to understand the content of the categories and to distinguish between the characteristics of different categories correctly, which is also referred to as response category appropriateness. The rating scale function was analyzed according to the following criteria. First, the category measure for each question should increase monotonically. Second, individual fit values over 1.5 for a rating on a scale such that the ideal value is 1.0 points suggest that the rating scale is not functioning effectively, and the corresponding categories should be merged (Linacre, 2006a). In cases where the response category did not increase monotonically, we planned to merge the categories to minimize this problem.

#### Separation Indices

In Rasch analysis, the standard error of measurement is calculated according to all proficiency levels apart from the sample group, and two separation indices are computed. These are indices of person and item separation, which are used to describe the reliability of the test in Rasch analysis. The larger the separation index, the greater the extent to which the test can distinguish the measurement function level. We used the following criteria for the person separation index: (a) 1.50 represents an acceptable level of separation, (b) 2.00 represents a good level of separation, and (c) 3.00 represents an excellent level of separation (Duncan et al., 2003). The item separation index and reliability use the same criteria and are interpreted the same as person separation index (Duncan et al., 2003).

The person separation reliability reported by most Rasch computer programs is calculated by subtracting observed variance of person ability measures to mean of squared standard errors of person ability measures from one. The formula (Wright and Masters, 1982) is:

$$\mathrm{𝑃𝑒𝑟𝑠𝑜𝑛}\,\mathrm{𝑆𝑒𝑝𝑎𝑟𝑎𝑡𝑖𝑜𝑛}\,\mathrm{𝑅𝑒𝑙𝑖𝑎𝑏𝑖𝑙𝑖𝑡𝑦}=1-\left[\frac{M\,S\,E_{p}}{S\,D_{P}^{2}}\right]$$

Where *MSE*_*p*_ is the mean square of the observed variance of person ability measures, and $S\,D_{P}^{2}$ is the mean squared standard errors of person ability measures.

The item separation reliability is calculated by the observed variance of item difficulty measures to mean of squared standard errors of item difficulty measures. The item separation index gives the test user an indication of how well items are separated by the persons taking the test. The formula for this index is (Wright and Masters, 1982):

$$\mathrm{𝐼𝑡𝑒𝑚}\,\mathrm{𝑆𝑒𝑝𝑎𝑟𝑎𝑡𝑖𝑜𝑛}\,\mathrm{𝑅𝑒𝑙𝑖𝑎𝑏𝑖𝑙𝑖𝑡𝑦}=1-\left[\frac{M\,S\,E_{i}}{S\,D_{i}^{2}}\right]$$

Where *MSE*_*i*_ is the mean square of the observed variance of item difficulty measures, and $S\,D_{i}^{2}$ is the mean squared standard errors of item difficulty measures.

## Results

### Item Fit

Table 2 shows the item fit statistics by order of entry into the model. There were no overfit items and no misfit items. Items with an MnSq value were above 0.5, and items with an MnSq value were below 1.7. Across all ten items, the range of infit MnSq values was from 0.71 to 1.25, whereas the range of outfit MnSq values was from 0.71 to 1.26. The overall fit statistics on the 10 items of the Rosenberg Self-Esteem Scale demonstrated a good fit.

### Item Difficulty

**FIGURE 1:**
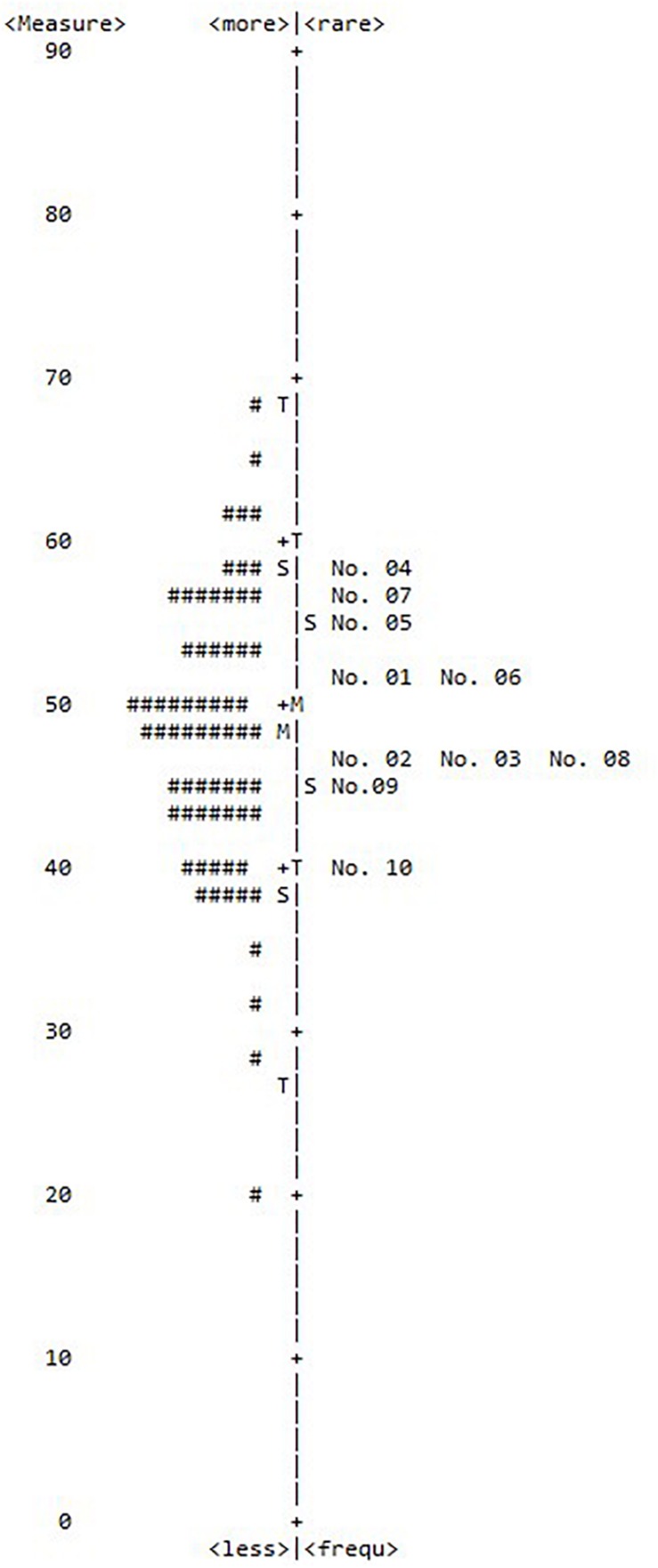
Item difficulty. Each (#) represents three individuals with ID; more, high person ability; less, low person ability; rare, high item difficulty; less, low item difficulty; +M, item mean; M, person mean; S = 1 *SD* from the mean; T = 2 *SD* from the mean.

Figure 1 presents a map of the individual proficiencies and item difficulties for the 10 items of the Rosenberg Self-Esteem Scale. More means high person ability, and less means low person ability. Rare means high item difficulty, and frequent means low item difficulty. The most difficult item was Item 4, and the easiest was Item 10. A total of 15 individuals with ID exhibited higher proficiency estimates than the ease of Item 4, and a total of 27 individuals with ID exhibited lower proficiency estimates than the difficulty of Item 10. The range of the respondents’ locations was wider than the range of the difficulty level of the items.

### Appropriateness of Rating Scale

**FIGURE 2:**
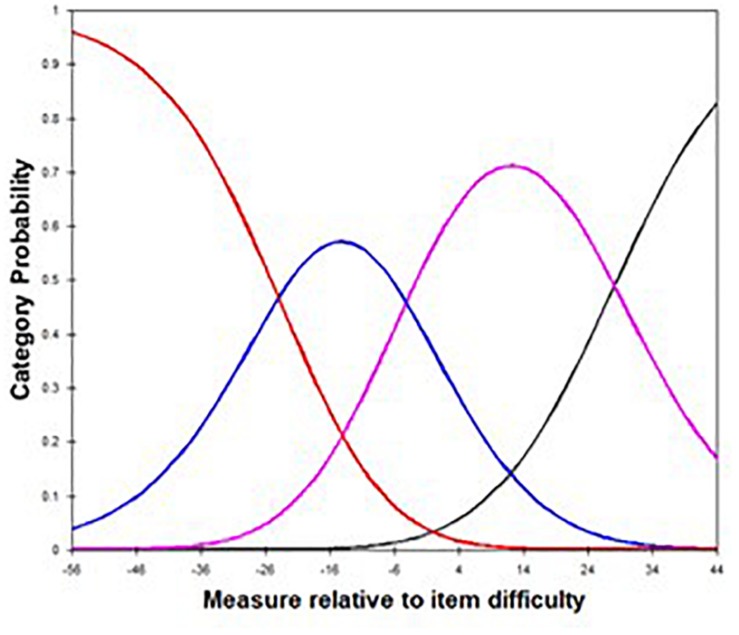
Category probability curve. Red line, Category 1; Blue line, Category 2; Pink line, Category 3; Black line, Category 4.

The rating scale of the Rosenberg Self-Esteem Scale was shown to be appropriate for individuals with ID. The results of the 4-point rating scale analysis are presented in Table 3 and Figure 2. This analysis of each aspect of the rating scale indicated that the Rosenberg Self-Esteem Scale was appropriately adapted for use with individuals with ID. The fit statistics were below 1.5 for each response category, and analysis of the scale threshold showed that increase along with increases in the response category.

**TABLE 3:** Rating scale analysis of 4-point scale (*N* = 223).

**Category level**	**Observed count**	**Observed count %**	**Observed average**	**Infit MNSQ**	**Outfit MNSQ**	**Structure calibration**
1	220	10	–17.41	0.94	0.96	−
2	831	37	–5.94	0.92	0.90	–23.95
3	1050	47	–5.94	0.88	0.89	–4.16
4	119	5	6.42	1.38	1.28	28.11

*MNSQ, mean square; Observed Count = the count of occurrences of this category; the expected MNSQ values for all categories are 1.0; Structure calibration is a calibrated measure of the transition from category below to this category, so the value of Category Level 1 cannot be calculated and is marked as none.*

### Separation Reliability

The person separation reliability value was 0.73, and the separation index was 1.66. The item separation reliability value was 0.76, and the separation index was 1.80. Thus, both separation indices represented an acceptable level of fit for the Rosenberg Self-Esteem Scale. The separation index and reliability of the Rosenberg Self-Esteem Scale were at an acceptable level for individuals with ID.

## Discussion

Although individuals with ID often participate in evaluations of their self-esteem, the validity of the most commonly used scale, the Rosenberg Self-Esteem Scale, using IRT has not previously been verified. While the validity of the Rosenberg Self-Esteem Scale has been extensively studied in the general population, the findings have not been entirely consistent. Some studies (Hagborg, 1993) have supported a one-dimensional scale structure, and another study partially supported the one-dimension (Corwyn, 2000). This study aimed to identify the item fit, item difficulties, and the appropriateness and reliability of the rating scale of the Rosenberg Self-Esteem Scale in individuals with ID using Rasch analysis.

In the current study, no misfit items were identified among the 10 items of the Rosenberg Self-Esteem Scale. A previous study with an elderly sample which used the Rasch analysis on the Rosenberg Self-Esteem Scale also showed appropriate fit for all 10 items with infit MnSq range from 0.80 to 1.24 and outfit MnSq range from 0.75 to 1.32 (Classen et al., 2007). Although we identified no misfit items on the Rosenberg Self-Esteem Scale, the possibility of misfits was considered. Items 1 (“I feel that I’m a person of worth, at least on an equal plane with others”) and 7 (“On the whole, I am satisfied with myself”) showed borderline levels of fitness. The MnSq values of these items were low. Specifically, the infit MnSq of Item 1 was 0.71, and the outfit MnSq was 0.71. The infit MnSq of Item number 7 was 0.72, and the outfit MnSq was 0.71. These values indicate that an item is redundant with other items (Hong et al., 2005) and suggest that further research is necessary to determine whether individuals with ID understand the meaning of these items differently from that of other items on the Rosenberg Self-Esteem Scale. Although unidimensionality of scale was supported through an analysis of the fit indices, a prospective study would further elucidate the interaction of individuals with ID’s characteristics and their understanding of item content.

The results of the item difficulty analysis were presented in the form of a figure comparing the locations of individuals with ID and the locations of items along the latent self-esteem dimension. As shown in Figure 1, the range of the respondents’ locations was wider than the range of difficulty levels of the items. The range of item difficulty was from 40.30 (Item 10) to 58.31 (Item 4). Additionally, the number of participants with high ability scores (above the most difficult item, namely Item 4) was 27 (16.3%), indicating that the items were not difficult for individuals with ID. A previous study which verified the Rosenberg Self-Esteem Scale using Rasch analysis (Classen et al., 2007) and reported that 73 elderly (7.4%) showed a ceiling effect. The results of this study show that the difficulty of the Rosenberg Self-Esteem Scale is appropriate for individuals with ID. However, the appropriateness of the scale may vary depending on the specific characteristics of the respondent. Future research should investigate whether the difficulty of the Rosenberg Self-Esteem Scale is also appropriate for younger people with ID, considering that this study collected data on participants who were at least 15 years old. Finally, Item 3 and Item 8 showed the same item difficulty (measure = 47.22), suggesting that only one of these items is necessary for discriminating level of self-esteem in individuals with ID.

The results of the analysis showed that the rating scale was appropriate, in that the fit indices for each response category were below 1.5 (Andrich, 1996). The range of infit MnSq according to category level was from 0.88 (category level 3) to 1.38 (category level 4), and outfit MnSq range was from 0.89 (category level 3) to 1.28 (category level 4), indicating that the conditions for appropriateness were satisfied by the Rosenberg Self-Esteem Scale, and the 4-point scale reflected the characteristics of the item responses. The probability curve (Figure 2) represents the probability of use of each response option. An ideal model is one in which the curve for each response option on the scale forms a bell-shaped curve, without overlap between the curves. The response probability curves for the Rosenberg Self-Esteem Scale showed that the rating scale was appropriate.

There are two types of separation index in Rasch analysis. Person separation reliability represents the same concept as Cronbach’s α. In the present study, the person separation reliability value for the Rosenberg Self-Esteem Scale in individuals with ID was 0.73, and the corresponding separation index was 1.66; the item separation reliability value was 0.76, and the corresponding separation index was 1.80. The separation index represents the extent to which the scale can distinguish each person or item. A separation index value of 1.5 represents an acceptable level of separation, and a value above 2.0 indicates a good level of separation (Fisher, 1992; Duncan et al., 2003). The separation indices for both person and item separation for the Rosenberg Self-Esteem Scale were identified as acceptable. The person separation index of 1.66 indicated that our sample could be separated into at least two statistically distinct groups according to self-esteem levels. However, the level of reliability in individuals with ID was lower than has been found in other samples. Internal consistency of 0.89 (Hagborg, 1993) was reported in a sample of adolescents, and 0.85–0.88 was reported in US students (Martín-Albo et al., 2007).

Having a scale that allows for assessments to meet scientific or practical needs is an essential precondition. If a valid measure does not exist, a new measure must be developed. The Rosenberg Self-Esteem Scale, a traditionally used measure of self-esteem, has been identified in the present study as possessing appropriate psychometric properties in individuals with ID. It may be argued that the results might not generalize to all individuals with ID, given that certain abilities were necessary to participate in the present research, such as language abilities or conceptual abilities. However, it is not the place of researchers to judge that people with ID should be excluded from participation in research; instead, they can take measures to ensure that people with ID are fully involved in the investigation and ensure that ethical practices are followed (Emerson et al., 2004). Beail and Williams (2014) report that the level of detail given in responses by people with ID is less rich than that of responses by the general population, but this does not mean that responses from the former group are less valuable.

This study is meaningful in that it confirms the psychometric properties of the Rosenberg Self-Esteem Scale in individuals with ID, but there are also some limitations. First, this panel survey that sampled only people with disabilities who were aged 15 and above. Therefore, there is a limit on the extent to which the findings can be generalized to individuals with ID aged under 15. A second limitation also arises from the nature of the sample. Data were collected from a total of 398 individuals with ID in the PSED; however, data from only 232 respondents were entered into the present analysis because the data from 166 respondents were not self-reported. This discrepancy indicates the need for the development of new tools to measure self-esteem in individuals with ID who may have difficulty understanding the content of scale items of the Rosenberg Self-Esteem Scale. Although we analyzed large survey data by using data from the PSED, this limits the representativeness of the sample regarding the population of individuals with ID. A third limitation was related to insufficient information about demographic characteristics. Within the population of people with ID, there are people with genetic problems, such as Down syndrome, who have other unique characteristics. The nature of the panel data used in this study does not allow us to have information about these genetic characteristics and the variety of differences in the sample. Further, this study did not compare the item difficulties estimated in individuals with ID with other samples to check whether the scale assesses the same construct across different subgroups.

## Conclusion

A positive view of research participation by people with ID is that their involvement in such studies represents a means of improving their lives, and in doing so, they also gain personal benefits, such as new experiences and improvements in their self-esteem (McDonald et al., 2013, 2015). In this study, the psychometric properties of the Rosenberg Self-Esteem Scale in individuals with ID were analyzed using the Rasch model. As part of the analysis, results indicating item fitness and difficulty, rating scale analysis, and reliability outcomes were derived. Based on the results, all 10 items of the Rosenberg Self-esteem scale showed appropriate levels of item fit, and the rating scale item difficulties and level of reliability were also found to be acceptable. The results of the item fitness test indicated that further tests are needed to confirm whether Items 1 and 7 overlap with other items. In conclusion, the reliability and validity of the Rosenberg Self-Esteem Scale for use with people with ID were confirmed through the application of the Rasch model to examine the psychometric properties of the items. Therefore, we conclude that the Rosenberg Self-Esteem Scale can be used to effectively evaluate the level of self-esteem of individuals with ID and in interpretive applications, such as the evaluation of the effectiveness of self-esteem programs.

## Data Availability

The datasets used and/or analyzed during this study are available from the corresponding author on reasonable request.

## Ethics Statement

Ethical review and approval was not required for the study on human participants in accordance with the local legislation and institutional requirements. The patients/participants provided their written informed consent to participate in this study.

## Author Contributions

J-YP made substantial contribution in drafting the manuscript. E-YP made substantial contribution in interpretation and analysis of the data and drafting the manuscript. Both authors read and approved the final manuscript.

## Conflict of Interest Statement

The authors declare that the research was conducted in the absence of any commercial or financial relationships that could be construed as a potential conflict of interest.

## Footnotes

1 https://edi.kead.or.kr/Index.do
